# Increased serum complement C3 and C4 concentrations and their relation to severity of chronic spontaneous urticaria and CRP concentration

**DOI:** 10.1186/1476-9255-10-22

**Published:** 2013-05-24

**Authors:** Alicja Kasperska-Zajac, Alicja Grzanka, Edyta Machura, Maciej Misiolek, Bogdan Mazur, Jerzy Jochem

**Affiliations:** 1 Clinical Department of Internal Diseases, Allergology and Clinical Immunology, ul. Ceglana 35, 40-952 Katowice, Poland; 2 Department of Internal Diseases, Dermatology and Allergology, Medical University of Silesia in Katowice, Katowice, Poland; 3 Department of Pediatric in Zabrze, Medical University of Silesia, Katowice, Poland; 4 Clinical Department of Otolaryngology in Zabrze, Medical University of Silesia, Katowice, Poland; 5 Department of Microbiology and Immunology, Medical University of Silesia, ul. Jordana 19, 41-808 Zabrze, Poland; 6 Department of Basic Medical Sciences, Medical University of Silesia, ul. Piekarska 18, 41-902 Bytom, Poland

**Keywords:** Complement, C3, C4, Acute phase response, Chronic urticaria

## Abstract

Chronic spontaneous urticaria (CU) is associated with activation of the acute phase response (APR). Nevertheless, APR-associated proteins have not been well characterized as potential biomarkers of the disease severity. To assess the pattern of complement proteins C3 and C4 – the acute phase reactants in patients with CU. C3, C4 and CRP concentrations were measured in serum of 70 patients showing different degrees of urticarial severity as well as in 33 healthy subjects. Serum C3 and C4 concentrations were significantly increased in CU patients as compared with the healthy subjects and exceed the normal lab range by about 5% and 10%, respectively. Significant differences were found between patients with mild and increased CU severity. In addition, significant correlations were observed between C3, C4 and CRP concentrations. More severe CU is characterized by higher production of C3 and C4 complements accompanied by parallel changes in CRP concentration.

## Introduction

Chronic spontaneous urticaria (CU) is associated with systemic inflammation and neuroimmunendocrine dysfunction, during which the acute phase response (APR) and coagulation/firbrinolysis processes are activated. Nevertheless, apart from IL-6 and C-reactive protein (CRP), other APR-associated proteins have not been well characterized as potential biomarkers of the disease severity [1-7]. It is known that the complement system is involved in mast cells activation in the course of CU [8]. The system is composed of many proinflammatory proteins. Among those, C3 is critical for activation of the complement system as a whole. On the other hand, C4 is the major protein of the classical cascade. They play an important role in the immune/inflammatory response and are upregulated during APR [9].

Nevertheless, APR pattern as well as the behaviour components of the complement system have not been well characterized in the disease.

Serum complement C3 and C4 concentrations were measured in CU patients with different degrees of urticarial severity in comparison with healthy subjects and normal population reference ranges.

## Methods

Seventy non-smoking patients with active chronic spontaneous urticaria of 13 months mean disease duration (range 4–40 months) without any concomitant physical urticaria were enrolled in the study.

Urticaria activity score (UAS) was estimated during four days and on the day of blood sampling: no wheals = 0, 1–10 wheals = 1, 11–50 wheals = 2, >50 wheals = 3) and pruritus intensity (no = 0, mild = 1, moderate = 2, severe = 3). UAS scores: daily (minimum = 0; maximum = 6) and four days by adding the daily score values (minimum = 0; maximum = 24). The UAS was graded as follows: 0–8 (mild), 9–16 (moderate) and 17–24 (severe). Our study comprised 20 patients with mild and 50 patients with moderate-severe utricaria symptoms as per the four days UAS. None of the examined subjects had taken oral corticosteroids or antidepressants within 8 weeks or antihistamines within at least 4 days before the study. Autologous serum skin test (ASST) and other investigations had been performed to exclude any known causes of the diseases or the concomitant diseases. Among those, routine dental and laryngological consultations were performed to exclude the infectious foci. The patients were divided into two groups: ASST-positive (n = 29) and ASST-negative (n = 41).

The control group consisted of 33 sex-, age- and BMI (<30) matched healthy subjects. None of the controls took any medication for at least 14 days before the study.

The Ethics Committee of the Medical University of Silesia approved of the study and written, informed consent was obtained from all the subjects participating.

### Blood collection

All blood samples were obtained between 7 and 9 a.m. by anticubital puncture.

### Assay of C3 and C4

Serum C3 and C4 concentrations were measured by the immunoturbidimetric method on Roche/Hitachi cobas c systems with a detection limit of 0.04 and 0.02 g/L, respectively. Normal range: (0.9 – 1.8 g/L) for C3 and (0.1 – 0.4 g/L) for C4.

### Assay of CRP

Serum CRP concentration was measured by the turbidimetric latex agglutination method (CRP-Latex, BioSystems SA, Barcelona, Spain) with a detection limit of 1.0 mg/l. Elevated serum CRP was defined as higher than 5.0 mg/l.

### Statistical analysis

Results are expressed as median and inter-quartile ranges. Because data were not distributed normally, nonparametric tests were used. Kruskal-Wallis variance analysis was used for screening differences between the groups. Mann–Whitney *U* test was used to compare data between the patient groups and the healthy controls. Spearman's rank test was used for correlations. The probability value of *P* < .05 was assumed significant.

## Results

### Serum C3, C4 and CRP concentrations in CU patients

**Figure 1:**
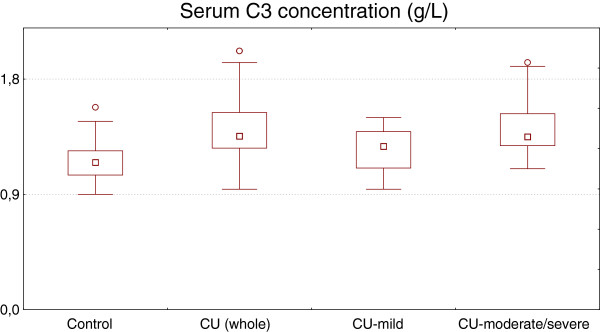
**Serum C3 concentration in healthy subjects and chronic urticaria (CU) patients with different disease activity.** CU (whole) vs. control, p < 0.0001; CU-moderate/severe vs. CUmild, p < 0.01; CU-mild vs. control, p > 0.05.

Serum C3 concentration was significantly higher in CU patients as compared with the healthy subjects (median: 1.36 *vs.* 1.15 g/L, p < 0.0001) (Figure 1). There were significant differences in serum C3 concentration between the CU patients with mild and moderate-severe symptoms (median: 1.28 *vs.* 1.42 g/L, p < 0.01) (Figure 1). About 95% had normal levels (range: 0.9–1.8 g/L) of serum C3, while 4 severe CU patients (5,7%) showed values above normal and none was below the limit.

Concentration of this complement in mild CU group did not differ significantly as compared with the healthy subjects and was within the normal lab range (Figure 1).

**Figure 2:**
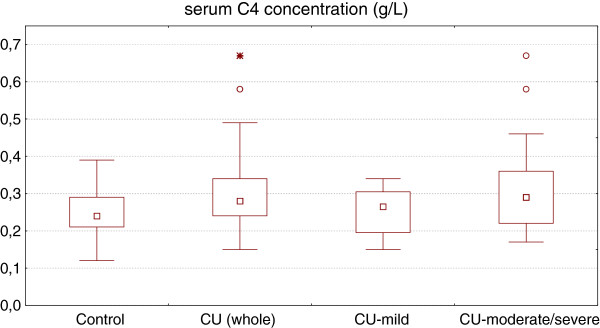
**Serum C4 concentration in healthy subjects and chronic urticaria (CU) patients with different disease activity.** CU (whole) vs. control, p < 0.05; CU-moderate/severe vs. CU-mild, p < 0.05; CU-mild vs. control, p > 0.05.

Serum C4 concentration was significantly higher in CU patients as compared with the healthy subjects (median: 0.28 *vs.* 0.24 g/L, p < 0.05) (Figure 2). There was a significant difference in plasma C4 concentration between the CU patients with mild and moderate-severe symptoms (median: 0.27 vs. 0.3 g/L, p < 0.05) (Figure 2). Only in 7 patients (10%) with severe CU, the C4 concentration exceeded the normal range upper limit value. The remaining patients had serum C4 concentration within the normal value (0.1–0.4 g/L). Concentration of this complement in mild CU group did not differ significantly as compared with the healthy subjects and was within the normal lab range (Figure 2).

**Figure 3:**
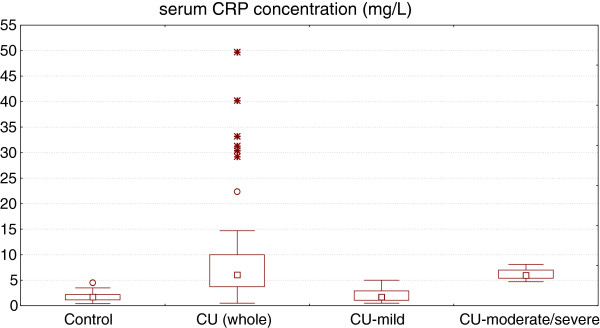
**Serum CRP concentration in healthy subjects and chronic urticaria (CU) patients with different disease activity.** CU (whole) vs. control, p < 0.0001; CU-moderate/severe vs. CUmild, p < 0.0001; CU-mild vs. control, p > 0.05.

Serum CRP concentration was significantly higher in CU patients as compared with healthy (median: 6.05 *vs.* 1.7 g/L, p < 0.0001). There was signifficant difference in CRP serum concentration between patients with mild and moderate-severe CU (median: 1.7 *vs.* 8.1 g/L, p < 0.0001), but not between mild CU and healthy subjects (Figure 3).

No significant differences in C3, C4 concentrations between ASST-positive and ASST-negative CU patients were observed (median: 1.33 *vs.* 1.39 and 0.32 *vs.* 0.28 g/L, respectively).

### Correlation between serum C3, C4 and CRP concentrations CU patients and in healthy subjects

There was signifficant correlation between serum C3, C4 and CRP concentrations in CU patients (r = 0.46, p = 0.000068 and r = 0.30, p = 0.011, respectively), but not in healthy subjects (r = 0.19, p = 0.3 and r = 0.21, p = 22, respectively). In addition, significant correlations were noted between circulating concentration of C3 and C4 (r = 0.55, p = 0.000001) in CU patients and in healthy subjects (r = 0.45, p = 0.008).

## Discussion

It has been demonstrated that IL-6 and CRP – biomarkers of APR correlate with severity and activity of CU [1,4,6,10]. In addition, there was a significant positive correlation between CU severity score and the mean platelet volume in ASST-positive patients, but not in ASST-negative CU patients [6].

In the present study, serum C3 and C4 concentrations were significantly higher in CU patients as compared with the healthy controls. There were significant differences between patients showing mild and more-severe degrees of urticaria severity, however not between mild CU and the control group. Such association might indicate a potential role of the complement components as a marker of the urticarial inflammation severity. On the other hand, C3 and C4 serum concentrations exceed the normal value only in about 5% and 10% patients with more severe CU. Thus, despite severe disease activity, C3 and C4 concentrations remains within the normal lab range in most patients. Such observations may restrict the clinical usefulness of such complements as biomarkers of the disease activity in clinical practice. Taking together the previous and the current studies, it seems that CRP is a more useful maker of CU severity than complements C3 and C4 [4]. Interestingly, significant correlations between C3, C4 and CRP were found in CU patients, but not in the healthy subjects.

Similarities are noted between various inflammatory biomarkers in CU, the complement components - C3, C4 and CRP share the common features belonging to APR proteins, which are synthesized mainly in liver, but there are also some differences in their behaviour during immune/inflammatory activation. In contrast to CRP, complement proteins C3 and C4 increase slower during APR - within days and persist longer - for several days [9].

It seems that elevated C3 and C4 are likely to result of increased synthesis in the liver in response to cytokines e.g. IL-1β, IL-6 or tumor necrosis factor (TNF), which are increased in active CU and are known to control synthesis of the APR proteins [9].

On the other hand, proximal and distal activation of the central component of the complement system, C3 is critical for activation of the system and leads to production of anaphylatoxins (C3a and C5a), which are able to stimulate secretion of various cytokines, including mentioned above and chemotaxis of different cells [11]. Interestingly, the C3aR is expressed on different cells associated with urticarial inflammation, including mast cells, basophils, eosinophils, neutrophils, monocytes [11]. In addition, the complement anaphylatoxins stimulate skin mast cells degranulation with release of histamine [12], vasodilation and increase the permeability of small blood vessels [11].

We do not know whether increased synthesis of C3 and C4 leads to enhanced generation of the active complements in CU. We can only speculate that at least one mechanism might be responsible for increased activation upregulated C3 and C4 in CU. It has been demonstrated that beta-tryptase, the major protease of human mast cells, can directly generate bioactive complements, such as C5a along with C3a and C4a. Interestingly, both beta-tryptase-generated C5a and C3a, but not C4a were able to activate human skin mast cells [13].

Our observation raises another important question: is the increased serum C3 and C4 concentrations in CU merely a simply biomarker of APR activation, reflected increased CU severity or they play some active role through the cleavage products in amplification urticarial processes. So far, available data regarding the behaviour of the complement system components in CU are scare and inconclusive [14]. It has been indicated that the complement cascade activation, in particular C5a is required for IgG-dependent histamine release form mast cells in CU [8]. This results indicate that the complement system may play an important role in urticarial processes and at least augments histamine release in autoimmune CU. Elevated C9 levels, but not other complements, including C3 and C4 have been reported in CU [15]. Also, in another study, complement C3 and C4 levels were within normal limits [16]. In contrast, Zamiri et al. reported both hypocomplementaemia, especially of C4, and positive ASST are common and often coexistent in CU [17]. Interestingly, C3 level was significantly higher in patients with CU with metabolic syndrome, and was directly correlated with a higher severity score and uncontrolled CU [14].

Further studies should examine the behaviour of active complement components in CU.

## Conclusions

CU patients with moderate-severe symptoms showed elevated C3 and C4 serum concentrations as compared with mild CU and healthy subjects.

The increased C3 and C4 production should complement the features of more severe CU and might support an involvement of APR and the complement system in urticarial processes. It it is known that corticosteroids reduce APR proteins production, we suggest then that CU patients with higher disease severity may require more extensive therapy, including corticosteroids or immunosuppressants. In addition antihistamines exerting anti-inflammatory effects seem to appear especially useful also in dosage higher than standard. High APR biomarkers concentration may be considered as a hallmark of enhanced systemic inflammation and one of the main indications for anty-inflammatory therapy.

Similarly to CRP, elevated serum C3 concentration predict strongly cardiometabolic risk [18]. This seems of special importance in long lasting, severe CU.

## Abbreviations

CU: Chronic spontaneous urticaria; APR: Acute phase response; CRP: C-reactive protein; C: Complement; ASST: Autologous serum skin test; UAS: Urticaria activity score.

## Competing interests

The authors declare that they have no competing interests.

## Authors’ contributions

AG and EM: collected samples and provided clinical data, contributed to data analysis and interpretation and wrote the manuscript. BM: performed the lab analysis, contributed to data analysis and interpretation. MM: provided clinical data, identified patients, contributed to statistcal data analysis and reviewed the manuscript. AKZ: conceived, designed and supervised the study and reviewed the manuscript. JJ: had the initial idea and helped write the manuscript. All authors read and approved the final manuscript.
